# Feasibility and preliminary efficacy of an online home-based functional exercise program for Parkinson's disease: a pilot study

**DOI:** 10.3389/fneur.2025.1591330

**Published:** 2025-06-23

**Authors:** Hyungwoo Lee, Hunyoung Ha, Heehyun Shin, Byungjun Park, Nyeonju Kang, Kiwon Park, Ryul Kim, Kyeongho Byun

**Affiliations:** ^1^Department of Human Movement Science, Incheon National University, Incheon, Republic of Korea; ^2^Division of Sport Science, Sport Science Institute & Health Promotion Center, Incheon National University, Incheon, Republic of Korea; ^3^Department of Biomedical and Robotics Engineering, Incheon National University, Incheon, Republic of Korea; ^4^Department of Neurology, Seoul Metropolitan Government - Seoul National University Boramae Medical Center, Seoul National University College of Medicine, Seoul, Republic of Korea

**Keywords:** Parkinson's disease, online home-based exercise, feasibility, exercise compliance rate, strength training

## Abstract

**Background:**

Parkinson's disease (PD) leads to motor and non-motor impairments, contributing to sarcopenia and reduced functional independence. While functional strength exercises can help manage these symptoms, adherence remains challenging, particularly in home-based setting.

**Objective:**

This pilot exercise intervention study aimed to evaluate the feasibility and efficacy of an 8-week Online Home-Based Exercise Program (OHEP), which provides easy-to-follow functional strength exercises for PD patients, enabling them perform these exercises safely and effectively at home.

**Methods:**

Fifteen patients with early-stage PD (Hoehn and Yahr Stage 1–2) participated in an 8-week exercise intervention, consisting of a 2-week in-person training followed by a 6-week online home-based exercise session using Zoom. The exercise regimen included softball, bodyweight, elastic band, and step box exercises targeting muscle strength, balance, and mobility. Feasibility was assessed through attrition rate, adherence rate, compliance rate, and safety. Efficacy was evaluated by examining changes in motor and non-motor symptoms, body composition, and physical performance.

**Results:**

Three participants withdrew from the study, resulting in an attrition rate of 20%. Feasibility was supported by a high adherence rate (median: 91%) and exercise compliance rates exceeding 93% across all exercise types. No adverse events reported. Among clinical outcomes, depressive symptoms significantly improved (Beck Depression Inventory, *p* = 0.011). Additionally, lower limb muscle function significantly improved, as reflected by a reduced time in the Five Times Sit-to-Stand test (*p* = 0.002). However, no significant changes were observed in other clinical or physical performance measures.

**Conclusion:**

These findings suggest that a short-term OHEP is feasible and safe intervention for PD patients, with potential benefits in improving depressive symptoms and physical function. However, further randomized controlled long-term studies are needed to better delineate the effects of this intervention in the management of PD.

**Clinical trial registration:**

https://cris.nih.go.kr, Identifier: KCT0008302.

## Introduction

Parkinson's disease (PD) is a chronic neurodegenerative disorder characterized by the dopaminergic neuron loss in the substantia nigra (1). It causes both motor symptoms (e.g., bradykinesia, muscular rigidity, resting tremor, and postural instability) and non-motor symptoms (e.g., cognitive and neuropsychiatric dysfunctions) (2). PD-related motor impairments increase energy expenditure, which can lead to weight loss, reduced muscle mass, and impaired mobility, contributing to sarcopenia (3, 4). Moreover, PD patients have a higher prevalence of sarcopenia than healthy older adults, which has been reported to contribute to changes in body composition and a decline in physical performance (5, 6). These motor and non-motor symptoms, along with sarcopenia, negatively impact activities of daily living and physical activity, ultimately reducing the quality of life for both PD patients and their caregivers (2, 7, 8). Therefore, interventions targeting both motor and non-motor symptoms in PD patients while preventing sarcopenia are crucial.

Exercise, particularly aerobic and strength training, has been widely reported to improve both motor and non-motor symptoms in PD, as well as sarcopenia-related outcomes (9–12). High-intensity aerobic exercise, in particular, has been shown to be a safe and effective intervention for rapidly improving both motor and non-motor symptoms in PD patients within a short period (e.g., 8 weeks) (13). However, to optimize functional independence and daily performance in PD patients, it is crucial to incorporate functional exercises—dynamic, multi-joint movements that facilitate energy transfer within the kinetic chain—alongside strength training. Despite these benefits, functional strength exercises pose participation challenging for PD patients. These include the need for supervision, injury concerns, and lack of exercise education (14). Moreover, the effects of short-term functional strength training in this population remain unclear.

Home-based exercise has been recognized as a viable alternative to traditional center-based programs for enhancing exercise adherence, addressing various challenges such as transportation, cost, efficiency, and potential health risks (15, 16). However, most home-based exercise interventions have primarily focused on aerobic exercises, while functional strength exercise-though beneficial-have seen limited application in home setting (17–19). Their implementation remains challenging due to the complexity of movement and the need for supervision, which may affect adherence in online formats (20). Furthermore, the lack of social interaction in online settings, a key factor in sustaining long-term participation, remains unresolved issue (18). Therefore, further research is required to determine the feasibility and broader applicability of online functional strength training programs for PD patients.

To address these challenges, the video conferencing platform ZOOM facilitates real-time monitoring, enabling individuals with PD to receive essential supervision and feedback during functional strength exercises while simultaneously fostering social interaction (15). Moreover, this approach retains the unique advantages of home-based exercise and is expected to enhance both feasibility and adherence in an online setting (21). However, despite its potential benefits, the feasibility and efficacy of this approach—particularly in evaluating whether individuals with PD can effectively perform various modalities of functional strength exercises in an online setting—remain inadequately explored. This assessment is essential for determining the long-term applicability of such exercises and their potential to mitigate both motor- and non-motor symptoms in PD.

Therefore, the current study aims to evaluate an 8-week Zoom-based Online Home-Based Exercise Program (OHEP) incorporating functional strength exercise for individuals with PD. The primary objective was to assess feasibility, including adherence, attrition, compliance, and safety measures. The secondary outcomes included motor and non-motor symptoms, body composition, and physical performance.

## Methods

### Participants

Patients with PD were recruited using consecutive sampling from the Department of Neurology at Inha University Hospital between March 2023 and May 2023. The diagnosis of PD was based on the UK PD Society Brain Bank criteria. Participants should be aged between 50 and 80 years; have no regular exercise habit; have a disease duration of less than 5 years; be classified as Stage 1 or 2 on the Hoehn and Yahr scale; and have been stable dopaminergic medication therapy for at least 3 months prior to enrollment. Exclusion criteria included the presence of neurological, orthopedic, or cardiac comorbidities that would contraindicate exercise, a Montreal Cognitive Assessment (MoCA) score below 18, and the use of antipsychotic medications.

This study was approved by the Clinical Trial Review Board of Inha University Hospital (2022-12-001) and registered at cris.nih.go.kr (KCT0008302). Written informed consent was obtained from all enrolled patients.

### Study design

This study was a single-center pilot trial designed to evaluate the feasibility and effectiveness of an OHEP for individuals with PD. The intervention lasted a total of 8 weeks and comprised two phases. The first phase consisted of a two-week in-person exercise training at the Incheon National University Sports Center, where participants received structured training on exercise techniques, the use of the Zoom platform, iPad usage, and the proper setup of their exercise space. Following the initial training phase, the second phase involved a six-week OHEP was conducted remotely via Zoom. Participants engaged in supervised virtual exercise sessions designed to improve motor- and non-motor symptoms while ensuring safety and adherence. The exercise regimen was structured to accommodate the remote format while maintaining participant engagement. The study design ensured that all remaining participant adhered to a structured exercise regimen tailored for remote delivery while maintaining feasibility and safety.

### Exercise program

The OHEP was delivered via Zoom and consisted of twice-weekly supervised sessions led by an exercise specialist who provided real-time guidance and feedback. The program aimed to improve physical function, mobility, and exercise adherence while minimizing barriers to in-person participation and addressing the motor symptoms characteristic of PD.

Each session followed a standardized structure comprising three phases: a 15-min warm-up, a 30-min main exercise phase, and a 10-min cool-down. The warm-up phase lasted for 15 min and included dynamic stretching, manual gymnastics, and balance exercise to prepare participants for the main session, with a focus on increasing mobility and reducing stiffness commonly observed in PD patients. The main exercise phase lasted for 30 min and incorporated multiple modalities including softball exercises, bodyweight exercises, elastic band exercises, and step box exercises. These exercises were selected to improve muscle strength, coordination, and functional mobility while considering the motor impairments associated with PD. The cool-down phase lasted for 10 min and included relaxation techniques and breathing exercise to aid recovery, enhance proprioception, and promote relaxation to mitigate muscle stiffness.

Participants were instructed to perform exercise in a safe environment. Caregivers were encouraged to be present when necessary to provide additional support. Exercise intensity was not actively regulated; however, participants were asked to report their perceived exertion using the Borg Rating of Perceived Exertion (RPE) scale (6–20) during each session, allowing the exercise specialist to monitor tolerance and ensure safety. Emergency contact procedures were in place to promptly address any adverse events. In addition, real-time monitoring was conducted by trained staff via the Zoom platform throughout the online sessions. Participants were instructed to report any adverse events, including falls, either during or between sessions. Although systematic fall diaries were not used, all sessions were directly supervised online, and no fall incidents were reported during the entire intervention period. A detailed breakdown of exercise type, repetitions, and target muscle groups (22) are provided in Supplementary Table 1.

### Measurements

#### Attrition rate, adherence rate, exercise compliance rate, and safety monitoring

To evaluate the feasibility of OHEP, four key components were assessed: attrition rate, adherence rate, exercise compliance rate, and safety monitoring. Adherence rate was calculated as the percentage of attended sessions out of the total scheduled session, with attendance recorded at each session throughout the eight-week program. In addition, the exercise compliance rate was evaluated using Zoom recording feature for session conducted during weeks 3–8. Each session was recorded, and two researchers systematically reviewed the footage to analyze participants' actual exercise performance compared to the prescribed regimen. The fidelity of the intervention was quantified using the following formula:

Exercise Compliance Rate (%) = (Actual Exercise Volume Performed/Prescribed Exercise Volume) × 100

This metric allowed for an objective assessment of how closely participants adhered to the planned exercise regimen and how effectively the exercise sessions were delivered in the remote setting. Additionally, it enabled an evaluation of the appropriateness and difficulty level of the prescribed exercise for individuals with PD, ensuring that the program was both assessable and suitably challenging for participants.

Safety monitoring was conducted through real-time observation during each exercise session and participants self-reporting. The exercise specialist closely monitored participants' physical condition throughout the session for any sign of discomfort, fatigue, or improper movement patterns. Participants were instructed to report any discomfort, pain, or adverse symptoms immediately during or after the sessions.

#### Motor and non-motor symptoms, body composition, and physical performance

All assessments were conducted before and after the intervention to evaluate changes in clinical outcomes, body composition, and physical performance. All assessments were performed while participants were in an off-medication state, requiring them to discontinue dopaminergic medication for at least 12 hours before testing.

The severity of PD symptoms was assessed using the Movement Disorder Society-Unified Parkinson's Disease Rating Scale (MDS-UPDRS), which evaluates both motor and non-motor aspects of the disease. Global cognitive function was evaluated using the Korean Dementia Rating Scale-2 (KDRS-2), which assess attention, executive function, construction, conceptualization, and memory (23). Depression was assessed using the Beck Depression Inventory (BDI). Anxiety was assessed using the Beck Anxiety Inventory (BAI). Apathy was assessed using the Apathy Scale (AS).

Body composition was measured using dual-energy X-ray absorptiometry (DEXA) (Lunar Prodigy Advance; GE healthcare, Madison, WI, USA) to assess total and regional muscle mass as well as body fat distribution. Physical performance was evaluated using three functional tests. Lower limb strength and functional mobility were assessed using the five times sit-to-stand test (5STS). Balance and gait function were evaluated using Times Up and Go (TUG) test. In addition, handgrip strength was assessed using a digital hand dynamometer (TK5401; Takei Scientific Instruments Co. Ltd., Niigata, Japan).

### Statistical analysis

All statistical analyses were performed using IBM SPSS Statistics (version 28.0, IBM Corp., Armonk, NY, USA). Due to the small sample size and non-normal distribution of the data, non-parametric methods were applied. Continuous variables were presented as medians and interquartile ranges (IQRs), and Shapiro-Wilk test was used to assess normality. Primary outcome measures (attrition rate, adherence rate, and exercise compliance rate) were analyzed descriptively. Difference in exercise compliance rate across exercise types were analyzed using the Friedman test, followed by Dunn's multiple comparison test with Bonferroni correction for *post-hoc*. Secondary outcomes were analyzed using the Wilcoxon signed-rank test was used to compare pre- and post-intervention values for motor- and non-motor symptoms, body composition, and physical performance. These included assessments of MDP-UPDRS scores, cognitive function (KDRS-2), depressive symptoms (BDI), anxiety (BAI), apathy (AS), body composition (DEXA-derived measures), and physical performance (5STS, TUG, and grip strength). All statistical tests were two-tailed, and significance was set at *p* < 0.05. For *post-hoc* comparisons, *p*-values were adjusted using Bonferroni correction.

## Results

### Participants' characteristics

A total of 15 were initially enrolled in this study. Among them, 12 successfully completed both pre- and post-intervention assessments according to a standardized protocol, resulting in an attrition rate of 20%. Three participants withdrew from the study: one due to difficulties in handing the device (Apple iPad) and using the Zoom platform, one due to health issues, and one due to scheduling conflicts with work commitments. The final analysis was conducted with 12 participants who completed the intervention. No adverse events were reported during the intervention.

The physical and demographic characteristics of the participants are summarized in Table 1. The median age was 65.50 years (IQR: 60.25, 73.75), with 33.3% of participants being male.

**Table 1:** Participants' characteristics.

**Variable**	**Value**
Participants (*n*)	12
Age (years)	65.50 (60.25, 73.75)
Sex [Male, *n* (%)]	4, 33.3%
Height (cm)	153.50 (149.20, 165.33)
Weight (kg)	59.55 (49.08, 69.60)
Education (years)	12 (9,12)

Data are presented as the median (interquartile range, IQR) unless otherwise stated.

### Feasibility assessments: adherence and exercise compliance rate

**Figure 1:**
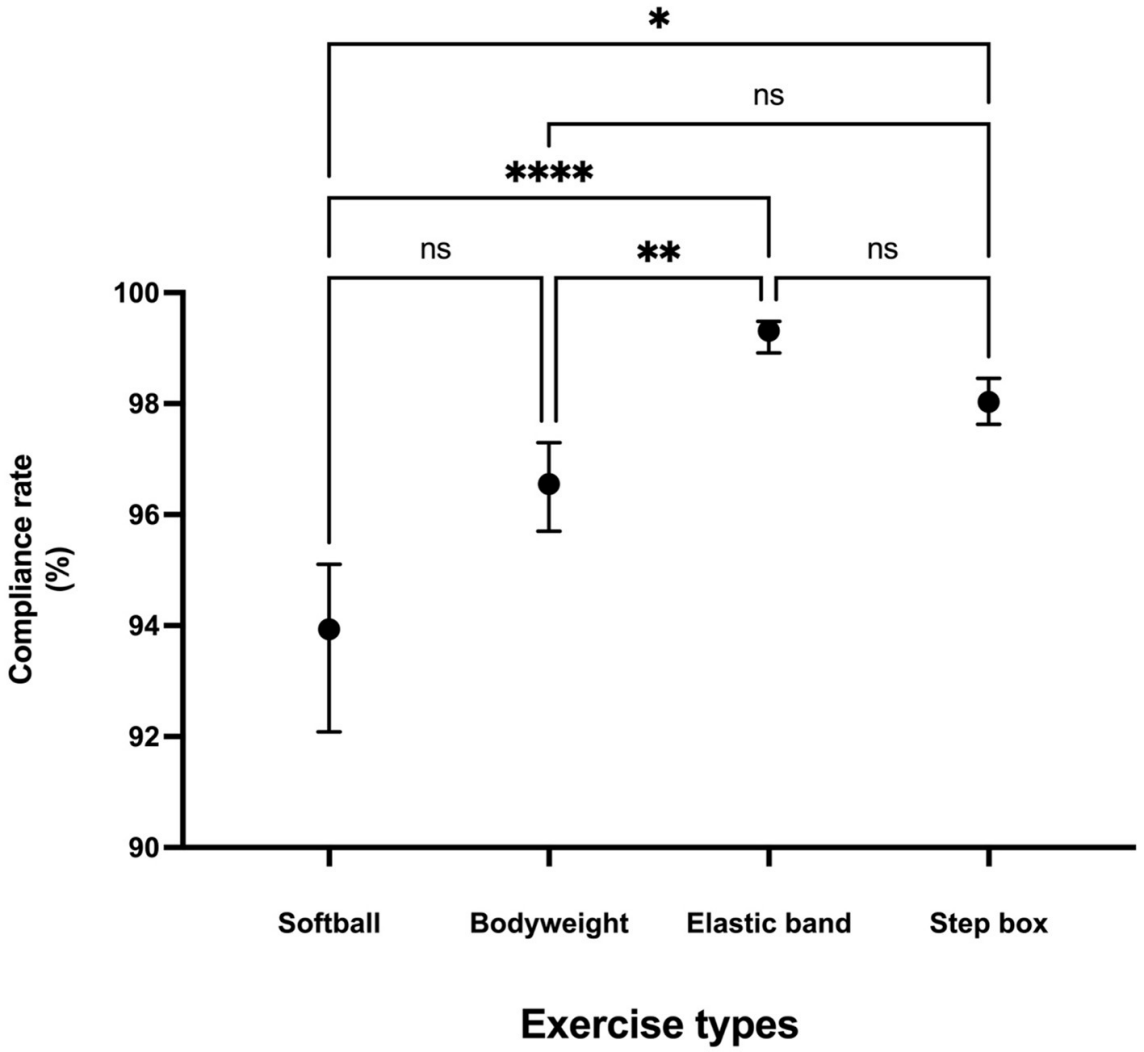
Exercise compliance rate across exercise types. Values are presented as median (interquartile range). Statistical significance was determined using Friedman test with Dunn's *post-hoc* test. ^*^p < 0.05; ^**^p < 0.01; ^***^p < 0.001.

The median attendance rate for the OHEP was 90.63% (ICR: 76.56, 98.44), indicating high adherence to the program. The exercise compliance rate was analyzed to assess participants' adherence to the intervention across different exercise types. The Friedman test revealed a significant difference in compliance rates among the four exercise types [χ^2^(3) = 28.30, *p* < 0.0001; Figure 1]. Among the exercise types, elastic band exercises demonstrate the highest compliance rate with a median of 99.31% (IQR: 98.91, 99.48), followed by step box exercise at 98.03% (97.63, 98.46), and bodyweight exercise at 96.55 % (IQR: 95.70, 97.30). The lowest compliance rate was observed for softball exercises, with a median of 93.94% (IQR: 92.08, 95.11). Dunn's *post-hoc* analyses revealed that the elastic band exercises had significantly higher compliance rates compared to softball (rank sum diff. = −32.00, *Z* = 5.060, *p* < 0.0001) and bodyweight exercises (rank sum diff. = −23.00, *Z* = 3.637, *p* = 0.0017). Step box exercises also showed a significantly higher compliance rate compared to softball exercises (rank sum diff. = −19.00, *Z* = 3.004, *p* = 0.0160). However, the remaining pairwise comparisons did not reach statistical significance (adjusted *p* > 0.05).

### Changes in motor and non-motor symptoms

There were no significant changes in MDS-UPDRS Part 1 (*Z* = −0.254, *p* = 0.799), Part 2 (*Z* = −1.328, *p* = 0.184), or Part 3 (*Z* = −1.034, *p* = 0.301). Similarly, no significant improvements were observed in the Korean Dementia Rate Scale-2 (KDRS-2) scores. Subscale scores for attention (*Z* = −0.879, *p* = 0.380), executive management (*Z* = −0.255, *p* = 0.799), structure (*Z* = 0.000, *p* = 1.000), conceptualization (*Z* = −0.674, *p* = 0.500), and memory (*Z* = −1.481, *p* = 0.139) did not show significant changes. The total score of KDRS-2 also remained unchanged (*Z* = −0.459, *p* = 0.646). Among neuropsychiatric symptoms, no significant differences were detected in BAI (*Z* = −0.979, *p* = 0.328) and AS (*Z* = −0.766, *p* = 0.443) scores. However, there was a significant reduction in BDI (*Z* = −2.558, *p* = 0.011; Table 2). The effect size for this change was large (*r* = 0.738).

**Table 2:** Changes in clinical outcomes before and after the intervention.

**Variable**	**Pre**	**Post**	***Z***	***p***	***r***
MDS-UPDRS part 1	5.50 (4.00, 9.25)	6.50 (5.25, 8.75)	−0.254	0.799	0.073
MDS-UPDRS part 2	4.50 (1.25, 6.75)	6.50 (2.00, 10.00)	−1.328	0.184	0.383
MDS-UPDRS part 3	24.00 (22.00, 25.38)	23.00 (21.00, 25.75)	−1.034	0.301	0.298
KDRS-2 attention	0.37 (−0.38, 0.70)	0.39 (0.00, 0.70)	−0.763	0.445	0.220
KDRS-2 executive function	0.45 (−1.54, 0.88)	0.23 (−0.97, 0.79)	−0.255	0.799	0.074
KDRS-2 structure	0.57 (0.27, 0.60)	0.57 (0.27, 0.60)	0.000	1.000	0.000
KDRS-2 conceptualization	0.45 (−0.29, 0.90)	0.45 (−0.29, 0.78)	−0.674	0.500	0.195
KDRS-2 memory	0.18 (−0.67, 1.12)	0.61 (−0.10, 1.19)	−1.481	0.139	0.428
KDRS-2 total score	0.58 (−0.16, 0.77)	0.58 (−0.16, 0.77)	−0.459	0.646	0.133
Beck depression inventory	9.00 (5.25, 13.25)	6.00 (1.75, 7.75)	−2.558	0.011^*^	0.738
Beck anxiety inventory	8.00 (4.00, 12.00)	3.50 (2.25, 10.50)	−0.979	0.328	0.283
Apathy scale	16.50 (8.00, 20.00)	15.00 (10.25, 16.75)	−0.766	0.443	0.221

Data are presented as the median (25%, 75%). ^*^p < 0.05 indicates statistical significance.

MDS-UPDRS, movement disorders society unified Parkinson's disease rating scale; KDRS-2, Korean dementia rating scale-2.

### Changes in body composition and physical performance

Significant changes were observed in body fat distribution, with reduction in arm fat percentage (*Z* = −2.228, *p* = 0.026) and leg fat percentage (*Z* = −2.043, *p* = 0.041). However, no significant differences were observed in total body fat percentage (*Z* = −1.156, *p* = 0.248) or trunk fat percentage (*Z* = −0.942, *p* = 0.346). Muscle mass remained stable throughout the intervention, with no significant changes in total muscle mass (*Z* = −0.392, *p* = 0.695), arm muscle mass (*Z* = −1.334, *p* = 0.182), and leg muscle mass (*Z* = −0.314, *p* = 0.754) or trunk muscle mass (*Z* = −0.078, *p* = 0.937). Lower limb muscle function, assessed by 5STS, showed a significant reduction in the time required to complete the test after intervention (*Z* = −3.061, *p* = 0.002). No significant changes were observed in TUG test (*Z* = −0.941, *p* = 0.347). Upper limb strength, assessed by relative grip strength, also did not show significant changes (*Z* = −0.445, P = 0.657; Table 3).

**Table 3:** Changes in body composition and physical performance before and after the intervention.

**Variable**		**Pre**	**Post**	***Z***	***p***	***r***
Body composition	BMI	23.23 (22.09, 29.05)	23.49 (21.69, 28.91)	−0.845	0.398	0.244
	Total muscle mass (g)	37454.00 (34200.00, 45360.25)	37606.00 (33761.50, 45514.00)	−0.392	0.695	0.113
	Arm muscle mass (g)	4162.00 (3514.25, 4737.25)	3831.50 (3431.00, 4463.75)	−1.334	0.182	0.385
	Leg muscle mass (g)	12494.00 (11025.50, 14619.75)	12060.00 (10976.75, 15060.00)	−0.314	0.754	0.091
	Trunk muscle mass (g)	18078.00 (17380.75, 21708.00)	18235.50 (17030.25, 21537.00)	−0.078	0.937	0.023
	Body fat (%)	32.95 (28.20, 39.23)	31.40 (26.95, 38.63)	−1.156	0.248	0.334
	Arm fat (%)	35.10 (27.13, 42.63)	36.00 (27.80, 42.85)	−2.228	0.026^*^	0.643
	Leg fat (%)	29.40 (24.73, 38.95)	28.30 (23.93, 37.85)	−2.043	0.041^*^	0.590
	Trunk fat (%)	35.95 (29.00, 41.60)	36.40 (26.88, 41.05)	−0.942	0.346	0.272
Physical performance	5STS (sec)	10.50 (9.56, 11.94)	8.35 (7.75, 9.40)	−3.061	0.002^*^	0.884
	TUG (sec)	7.70 (6.87, 8.45)	7.28 (6.42, 8.27)	−0.941	0.347	0.272
	Grip strength (%)	42.56 (33.58, 46.52)	42.39 (34.66, 46.85)	−0.445	0.657	0.128

Data are presented as the median (25%, 75%). ^*^p < 0.05 indicates statistical significance.

BMI, body mass index; 5STS, five times sit to stand; TUG, timed up and go test.

Among the observed outcomes, improvements in depressive symptoms and lower limb muscle performance appeared most pronounced. While these findings are promising, they should be interpreted cautiously given the exploratory nature of this pilot study.

## Discussion

This pilot study primarily aimed to evaluate the feasibility-specifically adherence, attrition, and compliance- of a short-term OHEP incorporating functional strength exercises for individuals with PD, with a secondary focus on exploratory clinical outcomes. The findings demonstrated high adherence (median attendance rate: 91%) and significant differences in exercise compliance among different modalities, with elastic band and step box exercises showing the highest compliance, while softball exercises had the lowest. These results suggest that structured, supervised functional strength exercises are feasible in an online home-based setting, through adherence varies by exercise type. Additionally, significant improvements were observed in depressive symptoms, as measured by the BDI, and lower limb muscle function, assessed by the 5STS test, suggesting potential psychological benefits and enhancement in physical function. However, these outcomes were exploratory in nature and, given the absence of a control group, should be interpreted cautiously rather than as definitive evidence of treatment efficacy.

The analysis of exercise compliance rates revealed significant differences among exercise type. *Post-hoc* tests indicated that compliance was the highest for elastic band exercises, followed by step box exercises, while softball exercises had the lowest adherence (Figure 1). One effective strategy to assist individuals with PD in overcoming motor symptoms is external and internal cueing to facilitate movement execution (24, 25). In this study, visual cues were implemented by having trainers perform the exercises alongside participants, while auditory cues were used to signal the start and end of each movement, and participants were instructed to count the repetitions aloud during execution. This structured approach likely contributed to the overall high exercise compliance observed in this study. Despite the generally high adherence across all exercise types, compliance with the softball exercises was relatively lower compared to other modalities. Softball exercise require mobility and stability of the trunk and shoulder joints, as well as coordinated movements of both hands, which can present additional challenges for PD patients. Due to dystonia, axial rigidity, soft tissue changes, and poor proprioception, PD patients often develop excessive trunk flexion, leading to muscle stiffness, weakened bimanual coordination, and reduced upper limb stability (26). Additionally, shoulder rigidity, frozen shoulder, akinesia, arm swing loss, and muscle weakness further impair shoulder function (27). These factors may have contributed to the slightly lower adherence observed in the softball exercise. Given these limitations, improving trunk and shoulder mobility and stability in PD patients may be beneficial for optimizing participation in upper-body exercises. Future programs may consider alternative upper-body exercises that require less trunk rotation and bilateral coordination, such as seated elastic band rows, unilateral pressing movements, or simplified object manipulation tasks. These adaptations may improve accessibility and adherence among individuals with PD-related axial rigidity and upper-limb motor dysfunction.

The clinical outcomes analysis revealed that, apart from improvements in depressive symptoms, no other variables showed significant changes. While this study did not observe significant changes in cognitive outcomes, this may be attributable to the relatively short intervention duration and the fact that cognitive enhancement was not a primary objective. Although the program did not include structured cognitive stimulation, it is possible that the addition of cognitively engaging elements may be beneficial in future studies specifically targeting cognitive improvements in PD. These findings should be interpreted with caution due to the small sample size, which may have limited the statistical power to detect meaningful effects. Additionally, the relatively short duration of the intervention is likely a contributing factor. Previous studies have suggested that home-based exercise programs with an intervention period of < 8 weeks or fewer than 30 total sessions may not be sufficient to induce significant improvements in overall motor symptoms in individuals with PD (28). Furthermore, compared to center-based programs, home-based settings may provide limited cognitive and emotional stimulation as well as fewer opportunities for social interaction, potentially reducing their effectiveness in addressing non-motor symptoms (29). Given these factors, the duration, environmental conditions, and social engagement aspects of the OHEP in this study may not have been adequate to produce significant improvements in broader clinical outcomes. Additionally, one participant withdrew early in the intervention due to difficulty operating the tablet device and using Zoom, despite an initial orientation session. This suggests the need to improve digital accessibility and usability among older adults with limited digital literacy. Future intervention may benefit from incorporating structured digital training, simplified interfaces, or caregiver involvement to support participant engagement and reduce early attrition.

However, consistent with previous research, the present study reinforces the notion that physical exercise exerts an antidepressant effects in individuals with PD (30). In particular, the selective improvements observed in depressive symptoms, but not in broader motor outcomes, may reflect differences in the time course and underlying mechanisms of adaptation. Mood-related benefits of exercise are thought to be mediated by relatively rapid neurochemical changes-such as increased brain-derived neurotrophic factor (BDNF) and monoamine neurotransmitter synthesis (e.g., serotonin, dopamine, norepinephrine)-that can occur within short-term interventions (31). In addition to neurochemical mechanisms, the structured and supervised nature of the intervention may have provided participants with a sense of routine, engagement, and external support, all of which can contribute to improved mood and psychological well-being in PD patients. In contrast, structural or neuromuscular adaptations required for motor improvement typically demand longer duration. Therefore, the short duration of the current intervention may have limited its capacity to generate broader motor outcomes. Future research should aim to address these limitations by optimizing intervention duration, enhancing environmental stimulation, and integrating interactive components to maximize the therapeutic benefits of OHEP.

The analysis of body composition revealed no significant improvement in muscle mass following the short-term OHEP, suggesting that the duration of the intervention was likely insufficient to induce measurable increases in muscle mass among individuals with PD. Given that PD is associated with progressive muscle atrophy and increased sarcopenia risk (3, 4), exercise interventions aimed at improving muscle mass may require longer durations and higher training volumes. Previous studies have indicated that for older adults, muscle hypertrophy typically requires a long-term exercise intervention of at least six months rather than a short-term program (32). Moreover, PD patients exhibit impaired neuromuscular activation and anabolic resistance (33–35), which could limit their potential for muscle mass gains in short-term interventions.

Regarding physical function, no significant improvements were observed in the TUG test or the relative grip strength, whereas the 5STS test showed a significant reduction in completion time, suggesting enhanced lower limb muscle function. This outcome aligns with the Specific Adaptation to Imposed Demand principle (36), which posits that physiological adaptations occur in response to specific training stimuli, and the transfer of training principle (37). This explains that practicing movement patterns similar to a given task can lead to improved performance in that task. Our exercise program emphasized functional lower limb exercises, such as squats and step box exercise (Supplementary Table 1), which may have specifically targeted neuromuscular activation in the lower extremities, leading to improvements in 5STS performance. However, these adaptations may not have been sufficient to elicit improvements in the TUG test, as it demands not only lower limb strength but also dynamic balance and gait coordination. Additionally, impaired neuromuscular plasticity and reduced motor unit recruitment in individuals with Parkinson's disease may further limit responsiveness to brief interventions targeting complex motor functions such as gait and balance (33, 34).

## Conclusion

Our findings demonstrated high adherence and compliance rates, particularly for elastic band and step box exercises, while also indicating improvements in depressive symptoms and lower limb muscle functions. However, no significant changes were observed in motor and cognitive function, muscle mass, or upper limb strength, suggesting that the intervention duration may not have been sufficient to induce broader physiological adaptations. Additionally, the inherent limitations of a home-based setting, such as reduced cognitive and social stimulation, may have influenced the outcomes. Despite these constraints, the results suggest that OHEP could serve as a viable approach to addressing specific functional and psychological aspects of PD. These findings suggest that home-based functional exercise may serve as a clinically useful and accessible intervention for individuals with mobility limitations or during circumstances requiring remote care, such as pandemics or other barriers to in-person rehabilitation. However, given the single-arm design, these findings should be considered preliminary. Future randomized controlled trials are warranted to assess efficacy and establish causal relationships. In addition, future studies should explore strategies to enhance its effectiveness, such as extending intervention duration, integrating progressive resistance training, and incorporating interactive elements to optimize engagement and long-term benefits. These may include hybrid delivery models combining in-person and online formats, social or peer support strategies, and gamification approaches to increase engagement.

## Data Availability

The raw data supporting the conclusions of this article will be made available by the authors, without undue reservation.
