# Supplementary material for: Feasibility and preliminary efficacy of an online home-based functional exercise program for Parkinson's disease: a pilot study
Source: Front Neurol. 2025 Jun 23;16:1591330. doi: 10.3389/fneur.2025.1591330 (PMC12229864; doi:10.3389/fneur.2025.1591330)
Supplement: Supplementary file 1 [file Table_1.docx]

**Supplementary Table 1. Structure of the Online Home-based Exercise Program**

| Exercise type and program | | Volume | Target muscles |
| --- | --- | --- | --- |
| Warm-up  (Dynamic stretching, Manual gymnastics, Balance exercise) | | 15 min | Stretching all muscle and joint |
| Softball exercise | Slow grip | 10 reps, 2 sets | FAM |
|  | Horizontal abduction |  | TRAP, LD, DELT, Core muscles, FAM |
|  | Up & downward rotation |  | TRAP, LD, DELT, Core muscles, FAM |
|  | Pitching |  | TRAP, LD, DELT, Core muscles, FAM |
|  | Shoulder press |  | TRAP, LD, DELT, Core muscles, FAM |
| Body weight exercise | Calf raises | 20 reps, 2 sets | GAS, SOL, FM, Core muscles |
|  | Squat |  | QF, HS, GM, GAS, SOL, Core muscles |
|  | Slow burpee | 10 reps, 2 sets | QF, HS, GM, IP, GAS, SOL, FM, Core muscles |
| Elastic band exercise | Band horizontal abduction with chair squat | 15 reps, 2 sets | QF, HS, GM, Core muscles, TRAP, LD, DELT |
|  | Band hip abduction | 10 reps, 2 sets | GM, Core muscles |
|  | Band horizontal abduction |  | Core muscles, TRAP, LD, DELT |
|  | Band chest press |  | PM, TB, Core muscles |
|  | Band bent over row |  | TRAP, LD, DELT, Core muscles |
|  | Band side lateral raise |  | TRAP, DELT, Core muscles |
|  | Band front raise |  | TRAP, DELT, Core muscles |
|  | Band biceps curl |  | BB, Core muscles |
| Step box exercise | Basic step | 10 reps, 2 sets | QF, HS, GM, IP, GAS, SOL, FM, Core muscles |
|  | Knee kick step |  | QF, HS, GM, IP, GAS, SOL, FM, Core muscles |
|  | Side step |  | QF, HS, GM, IP, GAS, SOL, FM, Core muscles |
| Abbreviation: QF, Quadriceps femoris; HS, Hamstrings; GM, Gluteal muscles; IP: Iliopsoas, GAS, Gastrocnemius; SOL, Soleus; FM, Foot muscles; PM, Pectoralis major; TRAP, Trapezius; LD, Latissimus dorsi; DELT, Deltoid; FAM, Forearm muscles; BB, Biceps brachii; TB, Triceps brachii. | | | |
